# Early fecal microbiota transplantation from high abdominal fat chickens affects recipient cecal microbiome and metabolism

**DOI:** 10.3389/fmicb.2023.1332230

**Published:** 2024-01-08

**Authors:** Jiani Song, Chaowei Luo, Zhijie Liu, Jingshou Liu, Li Xie, Xing Zhang, Zhuojun Xie, Xiangkun Li, Zheng Ma, Jinlong Ding, Hua Li, Hai Xiang

**Affiliations:** ^1^Guangdong Provincial Key Laboratory of Animal Molecular Design and Precise Breeding, School of Life Science and Engineering, Foshan University, Foshan, China; ^2^Guangdong Tinoo’s Foods Group Co., Ltd., Guangdong, China

**Keywords:** gut microbiota, cecal microbiome, chickens, fecal microbiota transplantation, fatty acids

## Abstract

Abdominal fat deposition (AFD) in chickens is closely related to the gut microecological balance. In this study, the gut microbiota from high-AFD chickens was transplanted into the same strain of 0-day-old chicks via fecal microbiota transplantation (FMT). The FTM from chickens with high AFD had no obvious effects on growth traits, adult body weight, carcass weight, abdominal fat weight, and abdominal fat percentage, but did reduce the coefficient of variation of AFD traits. FMT significantly decreased cecal microbiome richness, changed the microbiota structure, and regulated the biological functions associated with energy metabolism and fat synthesis. Additionally, the cecal metabolite composition and metabolic function of FMT recipient chickens were also significantly altered from those of the controls. Transplantation of high-AFD chicken gut microbiota promoted fatty acid elongation and biosynthesis and reduced the metabolism of vitamins, steroids, and carbohydrates in the cecum. These findings provide insights into the mechanisms by which chicken gut microbiota affect host metabolic profiles and fat deposition.

## 1 Introduction

With the development of breeding technology and nutrition level improvement, the growth and metabolic capacity of modern broiler chickens have rapidly improved but are accompanied by a significant increase in fat deposition that leads to a large amount of body fat deposition in broilers, especially in high-quality chickens (Abdalla et al., 2018). Studies have found that excessive deposition of abdominal fat not only affects the flavor components (Anthony, 1998; Dransfield and Sosnicki, 1999; Zhao et al., 2023) and reduces the taste of chicken, but also seriously reduces the slaughter rate of the fresh market population, reduces feeding efficiency, and increases environmental pollution through the production of large amounts of animal fat waste (Yan et al., 2015; Jiang et al., 2017). Therefore, exploring the regulatory mechanisms and factors influencing fat deposition in chickens is a research hotspot in this field.

Fat deposition in chickens is a physiological and biochemical process that is regulated by multiple factors that are affected by the body’s fat synthesis, catabolism, and transporter capacity, as well as linked to factors such as genetic regulation, nutritional level, and feeding mode (Chen et al., 2019; Xiang et al., 2021a; Nematbakhsh et al., 2021; Liu et al., 2023). Previous studies have shown that the genomes of gut microorganisms contain functional genes related to fat metabolism that are closely related to the host’s glucose metabolism, short-chain fatty acids (SCFAs) metabolism, and amino acid metabolism (Gill et al., 2006; Yan et al., 2018; Liu et al., 2022). The gut microbiome provides the energy needed for animal metabolism and fatty acids produced during lipid metabolism can promote fat deposition in intestinal epithelial cells (de Moreno de LeBlanc et al., 2018). Therefore, chicken fat deposition is closely related to the intestinal microecological balance (Ding et al., 2016). Fecal microbiota transplantation (FMT) has been shown to alter the diversity and species of intestinal flora by reconstructing the microecology of the host’s intestinal commensal bacteria, thereby modulating the nutrient absorption and metabolism of the host and altering the phenotypic traits of the host (Bäckhed et al., 2004; Ridaura et al., 2013; Qiao et al., 2014; Fu et al., 2023a). Previous studies have shown that FMT increases the abundance of probiotics such as *Lactobacillus* and the concentration of SCFAs enhances glycolipid metabolism and promotes the growth and development of chickens (Ma et al., 2023). However, there is a lack of studies on the effects of microbiota transplantation on the structure and metabolism of chicken intestinal microbiota and abdominal fat deposition.

To evaluate the effects of early high-fat microbiota transplantation on chicken abdominal fat deposition, intestinal microbiota was isolated from high AFD Qingyuan partridge chickens and transplanted into the same strain of Qingyuan partridge chicks at 0 days of age. The growth and development characteristics and abdominal fat deposition level of the recipient chickens were tracked and compared with those of the control group, and the profiles of 16S rRNA gene and metabolomics were used to evaluate the effects on the intestinal microbiota community and metabolic function.

## 2 Materials and methods

### 2.1 Chickens and sample collection

One hundred hens with similar body weights were randomly selected from the same batch of 140-day-old Qingyuan partridge chickens and raised under the same conditions. Traits such as body weight, body size, and abdominal fat weight were measured and the cecal contents were collected. By calculating the abdominal fat rate of each chicken, the cecal contents of the top 10 abdominal fat rate chickens with similar body weights were selected for bacterial suspension preparation as previously described (Yan et al., 2021). Briefly, 2 g of cecal content from each high abdominal fat chicken was mixed in equal amounts in 2 L 0.9% saline. Once the content was fully dissolved, it was incubated on ice for 10 min, and the supernatant was aspirated and filtered through medical sterile gauze and stored in sterilized EP tubes at −20°C.

Two hundred and forty 0-day-old chicks from the same batch of Qingyuan partridge hens were randomly selected and divided into two groups. One group was fed 1 mL of high-fat chicken bacterial suspension every day from the age of 0–9 days (GG), while the other group was fed saline under the same feeding conditions (EE). Both groups were then raised under identical conditions for 140 days.

### 2.2 Production performance and carcass character measurement

Each individual chicken was weighed weekly from 58 days old. Body slant length, keel length, crown height, shank length, shank circumference, chest width, chest depth, pelvic width, and waist circumference were measured about every 2 weeks for each individual chicken starting at 58 days. At 140 days, the slaughter weight and abdominal fat weight (AFW) were recorded, and abdominal fat percentage (AFP) was calculated.

### 2.3 Cecal content collection and 16S rRNA gene sequencing

After the chickens were slaughtered at 140 days, the contents of the cecum were immediately frozen in liquid nitrogen and stored at −80°C. Forty GG and EE individuals with similar body weights were selected for further analysis and the DNA of their cecal content was extracted using the QIAamp Fast DNA Stool Mini Kit. PCR amplification was performed using the universal amplification primer 341F/806R for the 16S rRNA gene V3-V4 region, and the PCR products were purified and used to construct high-throughput sequencing libraries using the TruSeq DNA PCR-Free Sample Preparation Kit, following the manufacturer’s protocols. Qualified libraries were subjected to high-throughput sequencing using PE250 on an Illumina NovaSeq 6,000 platform.

### 2.4 16S rRNA gene sequencing data processing

The sequenced data were processed using QIIME2 software (Bolyen et al., 2019). After filtering out the low-quality sequences, all the remaining high-quality reads were clustered into operational taxonomic units (OTUs) at a 97% similarity threshold. The OTU abundance and taxonomic classification from phylum to species were determined for each sample. The α-diversity metrics, including Shannon, Simpson, and Good’s Coverage indices, were calculated using QIIME2 software. A principal coordinate analysis (PCoA) plot based on Bray–Curtis dissimilarity was used to measure β-diversity. Differential analyses of taxa were performed using LEfSe analysis (Segata et al., 2011), and LDA scores>2.5 and *p* < 0.05 were selected as thresholds. Pearson’s correlation between differential microbes and AFW and AFP were then calculated. Microbial functions were predicted using PICRUSt (Langille et al., 2013), and principal component analysis (PCA) and DunnTest statistical analyses of the differential metabolic pathways (*p* < 0.05) between the groups were performed.

### 2.5 Metabolite profiling of cecal contents

Metabolite profiling of cecal contents was performed using UHPLC-Q/TOF-MS for 15 individuals with similar body weights from the GG and EE groups. Several quality control (QC) samples were prepared to monitor repeatability. The metabolites were separated using ultra-high performance liquid chromatography (Vanquish UHPLC, Thermo Fisher) and data was acquired using the Q Exactive™ HF platform (Thermo Fisher). Positive (ESI+) and negative (ESI-) modes were used to acquire MS data for alkali and acidic metabolites, respectively. The raw data were converted into the MzXML format using ProteoWizard software (Chambers et al., 2012). Peak identification, filtration, and alignment were performed using the XCMS package in R. A data matrix including mass-to-charge ratio (m/z), retention time, and intensity was constructed to identify the precursor molecules in the ESI+/− modes.

### 2.6 Differential metabolites identification and functional analysis

Subsequently, multivariate statistical analyses, including principal component analysis (PCA), partial least squares discriminant analysis (PLS-DA), and orthogonal PLS-DA (OPLS-DA) were conducted using the R package MetaboAnalystR. To construct a sample classifier for each group, support vector machines (SVM) and bagging models were applied to select the characteristic metabolites with the highest contribution to group differences. The prediction effect of the SVM model was represented by a ROC curve, which was depicted using the pROC package v2.0.1, in R. Metabolites with variable importance in projection (VIP) > 2, fold change >2, and *P*-adjust <0.05 were considered differential metabolites. The KEGG database was used to analyze the functional characteristics and classifications of the differential metabolites. The KEGG enrichment analysis was conducted using a t-test, and only pathways with *p* < 0.05 were considered significant.

### 2.7 Statistical analyses

All values were displayed as the mean ± SD. Significance tests for differences between groups were performed by one-way analysis of variance (ANOVA) for phenotypic data using SPSS 23 software. Significance tests for microbial diversity, differences in flora abundance, and differences in the degree of functional enrichment between groups were performed using R software based on ANOVA and *t*-tests, respectively. The *p*-values were adjusted by the false discovery rate (FDR) using the Benjamini–Hochberg method with the p.adjust function in R. All values with *p* < 0.05 indicated a significant difference, and *p* < 0.01 indicated a highly significant difference.

## 3 Results

### 3.1 Growth and abdominal fat deposition in FMT chickens

Considerable coefficients of variation (CV) were observed for both AFW and AFP in the GG and EE groups (Table 1). However, their body weight and carcass weight were similar.

**Table 1:** The descriptive statistics of growth traits and abdominal fat deposition of the donor and recipient chickens.

**Phenotypic traits**	**GG (*n* = 120)**		**EE (*n* = 120)**	
	**Mean ± standard deviation**	**CV(%)**	**Mean ± standard deviation**	**CV(%)**
BW (g)	1513.51 ± 144.70	9.56	1494.73 ± 171.94	11.5
CW (g)	1343.05 ± 138.14	10.29	1326.13 ± 160.19	12.08
AFW (g)	56.37 ± 17.33	30.74	52.65 ± 21.39	40.62
AFP (%)	3.72 ± 1.09	29.23	3.50 ± 1.36	38.79

BW, body weight; CW, carcass weight; AFW, abdominal fat weight; AFP, abdominal fat percentage; CV, coefficient of variation.

After transplantation of the high-fat chicken cecal bacterial suspension, the GG group consistently had slightly higher body weight than the EE group at 58–140 days (Supplementary Figure S1). No significant differences were observed in the body size traits between the two groups (*p* > 0.05; Supplementary Table S1). At 140 days, body weight, carcass weight, abdominal fat weight, and abdominal fat percentage were slightly higher in the GG group than in the EE group (*p* > 0.05); however, the CV for these traits in the GG group was lower than that in the EE group (Table 1). In particular, the variation in AFW (30.74% vs. 40.62) and AFP (29.23% vs. 38.79%) in the GG group decreased more significantly than that in the EE group (Table 1).

### 3.2 The effects of FMT on chicken cecal microbial diversity

**Figure 1:**
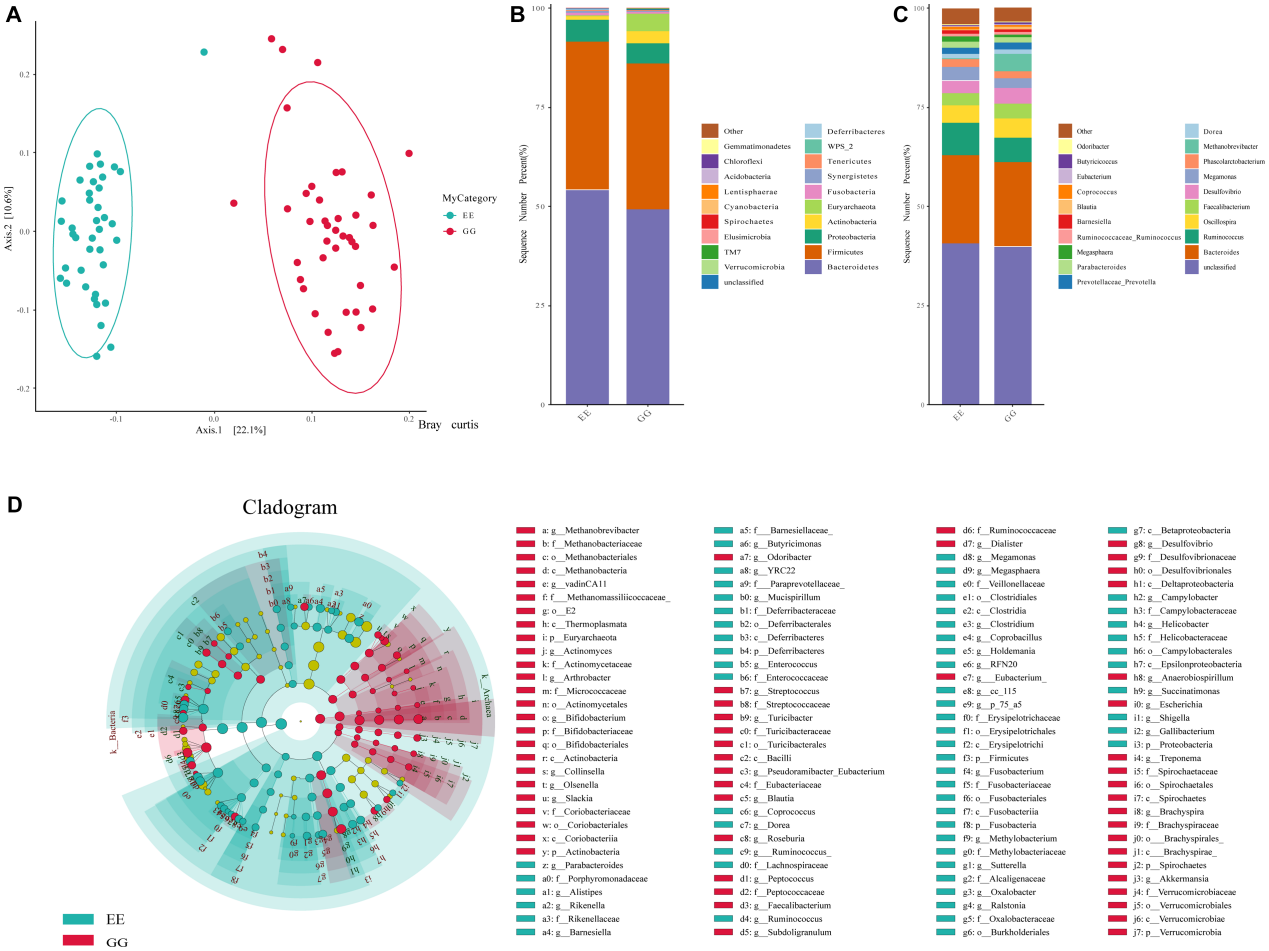
Gut microbial diversity and community between GG and EE chickens. **(A)** The PCoA analysis based on Bray Curtis statistic. **(B)** The comparison of the top 20 abundant phyla between GG and EE groups. **(C)** The comparison of the top 20 abundant genera between GG and EE groups. **(D)** Differential gut microorganism identified using LEfSe analysis.

An average of 104,621.2 high-quality reads per sample was obtained from the GG group, and there were 94,638.6 high-quality reads per sample from the EE group. The rarefaction curve of the observed OTUs for all samples plateaued, with an average Good’s coverage index of 0.9997 per sample (0.9991–0.9999), suggesting sufficient sequencing depth. A total of 1844 OTUs were identified for all samples, and similar taxonomic classification proportions were annotated for GG and EE individuals. Alpha diversity, including Shannon, Simpson, and Good’s coverage indices, suggested that early high-fat microbiota transplantation had significant negative effects on the chicken gut microbiome abundance (Supplementary Table S2). Beta-diversity analyses including Anosim, Permanova, and Permdisprevealed, also showed that different gut microbial communities in chickens with different AFD levels (Supplementary Table S3). The PCoA using the Bray Curtis test demonstrated a distinct separation between the GG and EE groups (Figure 1A), indicating a different gut microbial composition of early high-fat FMT chickens compared to control individuals.

### 3.3 The effects of FMT on chicken cecal microbiome taxa

The relative abundance of microbes was compared between GG and EE chickens to evaluate the effects of FMT on the chicken gut microbial composition. Among the top 20 dominant phyla in the GG and EE groups, *Bacteroidetes* and *Firmicutes* were the dominant taxa in both groups (Figure 1B), with a *Firmicutes*/*Bacteroidetes* ratio of 0.75 in the GG group, which was higher than that of 0.69 in the EE group. At the phylum level, the relative abundance of *Actinobacteria* and *Euryarchaeota* was higher in the GG group than in the EE group, whereas *Fusobacteria* and *Tenericutes* were less abundant in the GG group than in the EE group. Consistent with the results of phylum comparison, the dominant genera in both groups were *Bacteroides* and *Unspecified_Bacteroidales*, which were less abundant in the GG group than in the EE group (Figure 1C).

The LEfSe analysis of the taxonomic profiles clearly showed that the phyla *Actinobacteria* and *Euryarchaeota* and 34 genera, including *Methanobrevibacter*, *Desulfovibrio*, *Faecalibacterium*, *Blautia*, *Olsenella*, *Slackia,* and *Akkermansia*, as well as eight species, including *Bacteroides barnesiae*, *Bacteroides coprophilus*, *Subdoligranulum* var*iabile*, *Clostridium celatum,* and *Clostridium ruminantium* were significantly more abundant in the GG group than the EE group (Figure 1D and Supplementary Table S4). Additionally, six phyla (*Bacteroidetes*, *Lentisphaerae*, *Fusobacteria*, *Cyanobacteria*, *Tenericutes,* and *Elusimicrobia*) and 31 genera, including *Ruminococcus*, *Megamonas*, *Parabacteroides*m, *Methylobacteriaceae*, *Succinatimonas*, *Rikenella*, *Fusobacterium*; and 14 species, including *Anaerobiospirillum thomasii*, *Bacteroides plebeius*, *Megamonas hypermegale*, *Helicobacter equorum*, *Barnesiella viscericola,* and *Mucispirillum schaedleri* were significantly more abundant in the EE group than the GG group (Figure 1D and Supplementary Table S4).

Pearson’s correlation between the abundance of differential microbiota, AFW, and AFP showed that the abundance of the genus *Parabacteroides* decreased with an increase in AFW (*r* = −0.234, *p* < 0.05) and AFP (*r* = −0.231, *p* < 0.05), whereas the abundance of the genus *Clostridium* increased with AFW (*r* = 0.265, *p* < 0.05) and AFP (*r* = 0.263, *p* < 0.05) increased (Supplementary Figure S2). At the species level, the abundance of *A. thomasii* was negatively related to chicken AFW (*r* = −0.206, *p* < 0.05) and AFP (*r* = −0.242, *p* < 0.05) (Supplementary Figure S2).

### 3.4 Function capacities of the differential cecal microbes

**Figure 2:**
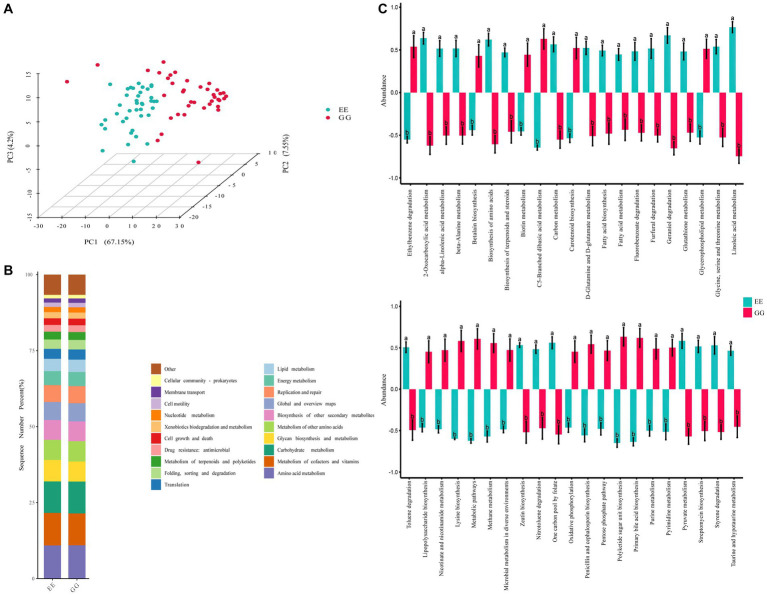
Predicted pathways of the differential microbiota in GG and EE chickens. **(A)** Principal component analysis (PCA) based on KEGG pathway enrichment. **(B)** The catalog of differential enriched KEGG pathways. **(C)** Statistically different metabolism pathways between GG and EE groups. Different letters indicates *P* < 0.05.

The PCA of the KEGG pathways for differential microbiota showed significant differentiation between the GG and EE groups, suggesting that early microbiota transplantation from high-fat chicken donors led to significant changes in the functional capacities of the recipient chicken gut microbiome community (Figure 2A). The differential functional pathways between the GG and EE groups were annotated to the L2 level pathways, and the results showed that the differential functional pathways were mainly related to 10 L2 level pathways of the catalog metabolism, including global and overview maps, amino acid metabolism, chemical structure transformation maps, lipid metabolisms, carbohydrate metabolism, biosynthesis of other secondary metabolites, and so on (Figure 2B and Supplementary Table S5). Specifically, 42 KEGG pathways were differentially expressed (*p* < 0.05) between the GG and EE chickens (Figure 2C), among which 19 pathways were upregulated in the GG group, including the biosynthesis of amino acids, D-glutamine and D-glutamate metabolism, glycerophospholipid metabolism, methane metabolism, metabolic pathways, primary bile acid biosynthesis, urine metabolism, and pyrimidine metabolism. There were 23 metabolic pathways that were down-regulated in the GG group, including alpha-linolenic acid metabolism, linoleic acid metabolism, biosynthesis of terpenoids and steroids, carotenoid biosynthesis, and fatty acid metabolism.

### 3.5 Early FMT of chicken cecal content alters metabolite composition

**Figure 3:**
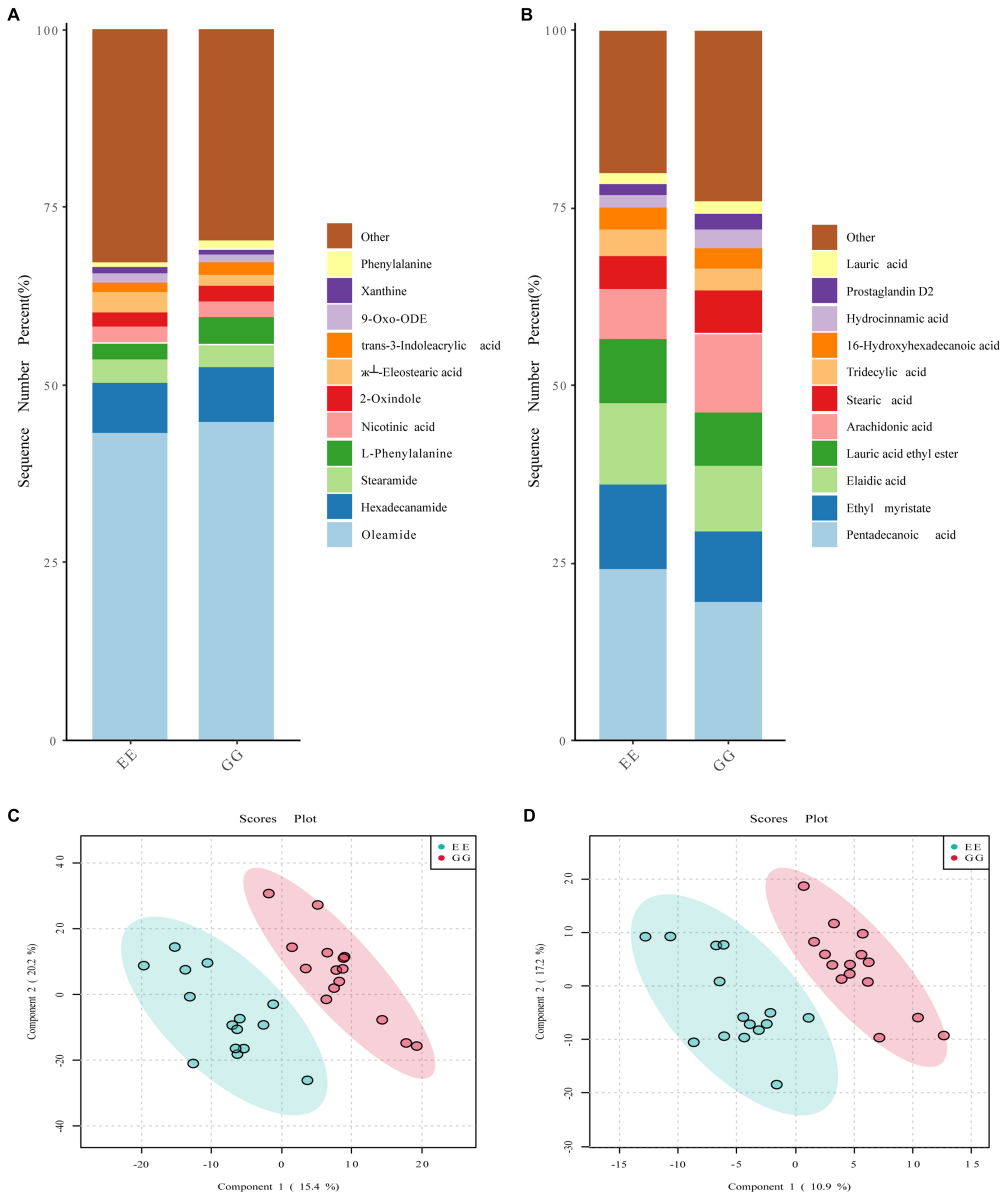
Metabolic profiles affected by the early transplantation of the high-fat chicken gut microbiota. The comparison of the top 10 metabolites identified in **(A)** positive ion mode and **(B)** negative ion mode between GG and EE groups. The PLS-DA score plots of **(C)** positive ion mode and **(D)** negative ion mode metabolites.

The signal drift recorded by the QC samples was stable, and all samples were closely clustered (Supplementary Figure S3), suggesting high repeatability of the experiment. A comparison of the dominant metabolites between the GG and EE groups showed similar metabolite compositions between these two groups. In the positive ion mode, the top 10 metabolites in both groups were oleamide, L-phenylalanine, trans-3-indoleacrylic acid, xanthine, l-esterearic acid, 9-oxo-ODE, 2-oxindole, nicotinic acid, stearamide, and hexadecanamide (Figure 3A). The top 10 metabolites in negative ion mode were prostaglandin D2, hydrocinnamic acid, 16-hydroxyhexadecanoic acid, tridecylic acid, stearic acid, arachidonic acid, lauric acid ethyl ester, elaidic acid, ethyl myristate, and pentadecanoic acid (Figure 3B). However, the PLS-DA results showed that individuals in the GG and EE groups were distributed in significantly different regions (Figures 3C,D), suggesting that the metabolite compositions of the cecal content of the two groups were significantly different. The clustering heatmap of all metabolites showed that there were a large number of metabolites that differed between the GG and EE groups in both the positive and negative ion modes (Supplementary Figure S4). Furthermore, an SVM classifier was used to evaluate the diagnostic value of the differential metabolites between the GG and EE groups. Among the top 15 characterized metabolites in the positive ion mode, 2-amino-1,3,4-octadecanetriol, +/−56- +/−56-EET ethanolamide, YMK, coniferin, 9-KODE, 1-methylxanthine, *N*-morpholinocarbonyl-4- morpholinosulfonylbenzamide, VLK, and 3-hydroxy-3, 4-bis4-hydroxy- 3-methoxyphenylmethyloxolan-2-one were more abundant in the GG group, whereas shikonin, 3-acetoxyurs-12-en-23-oic acid, nitrosoheptamethyleneimine, (б└)17(18)-EpETE, and 13,14-dihydro-15-keto prostaglandin A2 were more abundant in the EE group (Supplementary Figure S5). Among the top 15 characterized metabolites in the negative ion mode, cer-NS d181/160, 5-tert-butyl-2-methyl-*N*-5-methyl-3-isoxazolyl-3-furamide, tetradecanedioic acid, N-5-trifluoromethyl-2-pyridinylbenzenesulfonamide, 3-phenoxybenzoic acid, and dopamine HCl were higher in the GG group, while PE130/150, 3-hydroxybutyric acid, lysoPC 204, O1-4-chlorobenzoyl-4-nitrobenzene-1-carbohydroximamide, β-D-glucopyranuronic acid, cyclocytidine, 4-hydroxy-3-methylbenzoic acid, LPA 221, and 2,6-dihydroxybenzoic acid were higher in the EE group (Supplementary Figure S6). Group predictions using the screened characterized metabolites in both modes complied with an area under the receiver operating curve (AUC) of 0.932 (Supplementary Figure S5) and 0.952 (Supplementary Figure S6), further suggesting that the metabolic compositions were different between the GG and EE chickens.

### 3.6 Differential metabolites and functions affected by early FMT

The OPLS-DA analysis of metabolite expression was conducted to screen for important characterized metabolites, followed by an ANOVA significance test. The results showed that there were 24 significantly different important metabolites in the positive ion mode, including morpholine carbonyl-4-morpholine sulfonyl benzoamide, VLK, GLK, 2-amino-1.3.4-octadecanetriol, N-desmethyl sildenafil were significantly higher in the GG group; while 3-acetoxyurs-12-en-23-oic-acid, 2-{[4-(tert-butyl)benzyl]thio}-4-methylquinoline-3-carbonitrile, metanenhrine L-threonine, 13,14-dihydro-15-keto-prostaglandin A2, shikonin, L-saccharopine, DL-carnitine, deoxyinosine, and homo-gamma-linolenic acid (C20:3) were significantly higher in the EE group (Table 2). Five significant and important metabolites were present in the negative ion mode, including cer-NS (d18:1/16:0) and N-[5-(trifluoromethyl)2- pyridinyl] benzenesulfonamide, which were significantly higher in the GG group than in the EE group, and 3-hydroxybutyric acid, O1-(4-chlorobenzoyl)-4-nitrobenzene-1-carbohydroximami, and cyclocytidine, which were significantly lower in the GG group than in the EE group (Table 2).

**Table 2:** Differential metabolites affected by the early transplantation of high-fat chicken gut microbiota.

**Ion mode**	**Name of metabolite**	**VIP**	***P-*value**	**FDR**
Positive ion mode	2-{[4-(tert-butyl)benzyl]thio}-4-methylquinoline-3-carbonitrile	2.8165	0.0000	0.0073
	3-Acetoxyurs-12-en-23-oic acid	2.7475	0.0000	0.0073
	*N*-(morpholinocarbonyl)-4-(morpholinosulfonyl)benzamide	2.8048	0.0000	0.0077
	13,14-dihydro-15-keto Prostaglandin A2	2.5734	0.0001	0.0148
	L-Threonine	2.5471	0.0001	0.0148
	Metanephrine	2.5795	0.0001	0.0148
	VLK	2.5679	0.0001	0.0148
	GLK	2.5011	0.0001	0.016
	Shikonin	2.5133	0.0001	0.016
	2-Amino-1,3,4-octadecanetriol	2.3726	0.0002	0.0174
	L-Saccharopine	2.4868	0.0002	0.0174
	DL-Carnitine	2.4436	0.0003	0.024
	Deoxyinosine	2.4257	0.0005	0.0367
	Homo-Gamma-Linolenic Acid (C20:3)	2.3478	0.0005	0.0367
	*N*-Desmethyl sildenafil	2.3057	0.0005	0.0367
	Thiamine monophosphate	2.3413	0.0006	0.0367
	YMK	2.2882	0.0006	0.0367
	L-Dopa	2.2825	0.0007	0.0371
	*N*-Acetyl-L-glutamic acid	2.2775	0.0006	0.0371
	2-benzylideneindan-1-one	2.2675	0.0008	0.0394
	Dl-3-Hydroxynorvaline	2.2997	0.0008	0.0394
	L-(+)-Citrulline	2.2992	0.0008	0.0394
	SPK	2.2439	0.0008	0.0394
	Melanin	2.2165	0.0011	0.049
Negative ion mode	3-Hydroxybutyric acid	2.9243	0	0.01
	O1-(4-chlorobenzoyl)-4-nitrobenzene-1-carbohydroximamide	2.9571	0	0.01
	Cer-NS(d18:1/16:0)	2.7258	0.0001	0.0179
	*N*-[5-(trifluoromethyl)-2-pyridinyl]benzenesulfonamide	2.8169	0.0001	0.0179
	Cyclocytidine	2.5086	0.0004	0.0377

**Figure 4:**
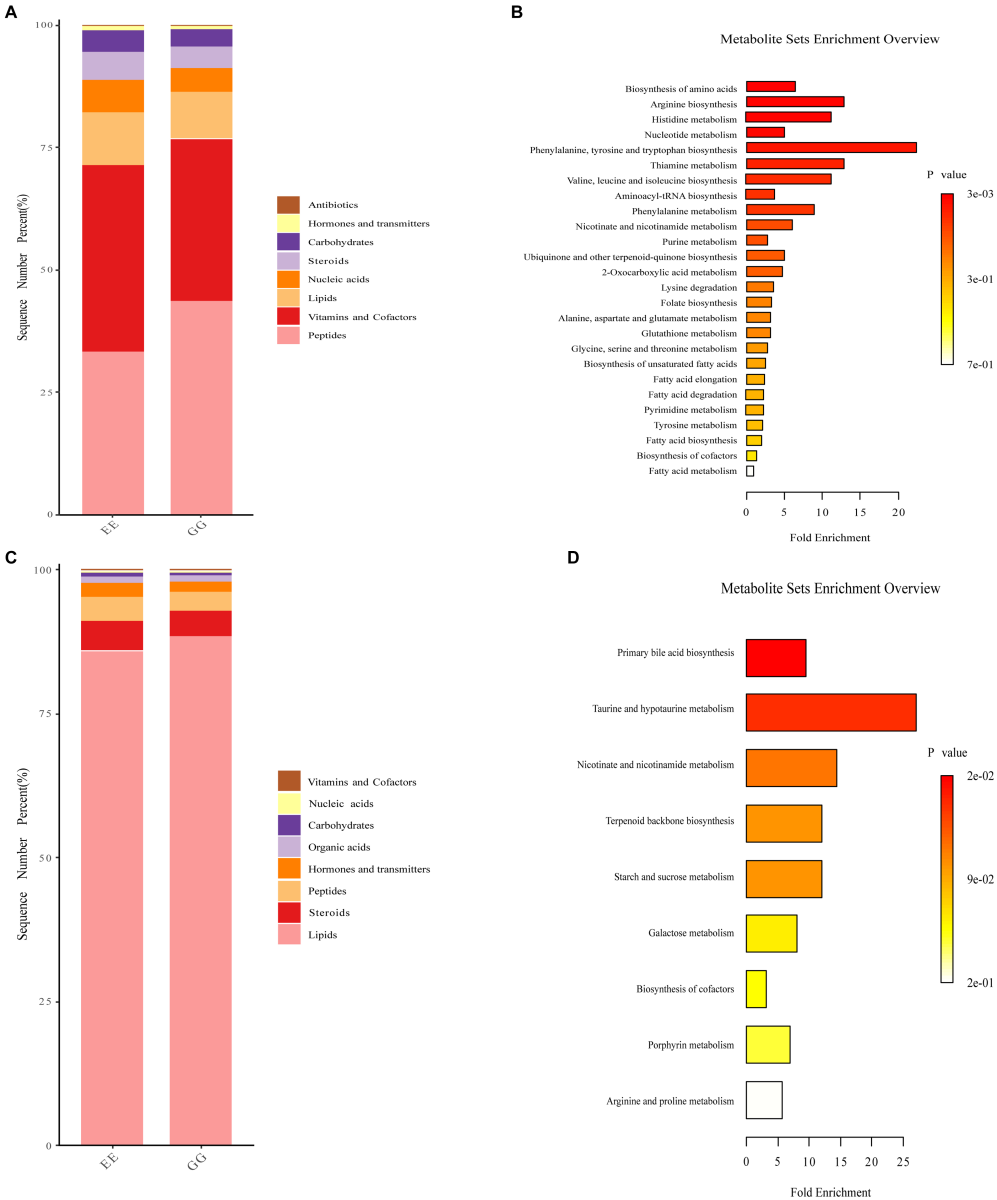
Functional annotation of differential metabolites in GG and EE chickens. Accumulation diagram of differential biological roles of metabolites enriched in **(A)** positive ion mode and **(B)** and negative ion mode mass spectrometry analysis. The differential pathways enriched by **(C)** positive ion mode and **(D)** negative ion mode metabolites.

Biological function analysis showed that peptide function was higher, while vitamins and cofactors, nucleic acids, steroids, and carbohydrates were lower in the positive ion mode metabolites identified in the GG group (Figure 4A). We also found that lipid function was higher, and steroids, hormones, and transmitters were lower for negative ion mode metabolites in the GG group (Figure 4B). KEGG pathway enrichment of the differential metabolites between the GG and EE groups showed that positive ion mode differential metabolites were enriched in arginine and proline metabolism, histidine metabolism, alanine, aspartate, and glutamate metabolism; thiamine metabolism, nicotinate and nicotinamide metabolism; fatty acid elongation in the mitochondria, fatty acid biosynthesis, fatty acid metabolism, and 23 other differential enrichment pathways (Figure 4C). Nine metabolic pathways were enriched in negative-ion mode differential metabolites, including primary bile acid biosynthesis, taurine and hypotaurine metabolism, terpenoid backbone biosynthesis, galactose metabolism, nicotinate and nicotinamide metabolism, arginine and proline metabolism, and starch and sucrose metabolism (Figure 4D).

## 4 Discussion

Abdominal fat deposition is important for improving chicken production efficiency and meat flavor. The Qingyuan partridge chicken is a typical local breed of slow and high-quality yellow-feathered broilers that are usually slaughtered and marketed when they reach sexual maturity at 140 days of age. Therefore, excessive abdominal fat deposition is a serious problem in Qingyuan partridge chicken production and was confirmed by the high AFW and AFP of both donor and recipient chickens in this study. Early microbiota transplantation from high-fat chickens did not affect growth traits, body weight, carcass weight, and body size of recipient Qingyuan partridge chickens but did greatly reduce the coefficient of variation of abdominal fat deposition traits, providing evidence that a change in the bacterial colony structure is important in the regulation of the abdominal fat deposition capacity of chickens.

Extensive studies have shown that the abundance and structure of gut microbiota are closely related to abdominal fat deposition in chickens (Ding et al., 2016; Wen et al., 2019). Based on our previous studies that reported the involvement of gut microbiota in the regulation of abdominal fat deposition in Qingyuan partridge chickens (Xiang et al., 2021b), the present study further showed that early microbiota transplantation from high-fat chickens significantly reduced the diversity of the microbiota in recipient chickens, which is consistent with the hypothesis that gut microbial abundance is negatively correlated with the level of abdominal fat deposition in chickens.

*Actinobacteria* and *Euryarchaeota* were the dominant cecum microbes at the phylum level; their abundance increased in recipient chickens after transplantation, whereas that of *Bacteroidetes*, *Lentisphaerae*, *Fusobacteria*, *Tenericutes*, and *Elusimicrobia* decreased. Previous studies reported that the biological functions of *Actinobacteria* and *Euryarchaeota* promoted fat deposition in chickens (Bäckhed et al., 2004). Torok et al. (2011) found that *Firmicutes*, *Bacteroidetes,* and other dominant taxa were correlated with broiler production performance, while Ding et al. (2016) reported that the abundance of *Fusobacteria* and their related genera and species in the feces and gut content of high-AFD chickens was lower than that of low-AFD chickens. In the gut biota of both obese and obese mice, the abundance of *Bacteroidetes* was significantly lower and the diversity of the biota was significantly reduced when compared with that of the normal group (Ley et al., 2006). Additionally, the relative abundance of *Firmicutes* and *Proteobacteria* increased and the abundance of *Bacteroidetes* decreased in the intestinal tract of pups after FMT (Tuniyazi et al., 2022).

In the present study, early microbiota transplantation from high-fat chickens increased the abundance of 34 genera in Qingyuan partridge chicken recipients, including *Methanobrevibacter*, *Desulfovibrio*, *Faecalibacterium*, *Blautia*, *Olsenella,* and *Slackia*. A positive correlation between *Methanobrevibacter* and body weight in children has been reported (Mbakwa et al., 2015), and we also found that the abundance of both *Methanobrevibacter* and *Mucispirillum schaedleri* were significantly correlated with chicken fat deposition (Wen et al., 2019). A previous study reported that a high-fat diet in normal mice resulted in weight gain, and susceptibility to obesity was closely related to *Desulfovibrio* and other gut microbiota, which may be involved in lipid metabolism disorders (Hong et al., 2021). Oral application of a complex polysaccharide to a high-fat diet for 6 weeks increased the abundance of *Bifidobacteraceae* and significantly decreased *Desulfovibrio* abundance in the guts of mice, suggesting the facilitation of fat deposition in *Desulfovibrio* (Sha et al., 2018). Both *Olsenella* and *Slackia* were more abundant in fat Tiannong partridge chickens than in lean ones, which were suggested to possibly enhance energy capture and positively contribute to fat deposition (Xiang et al., 2021b). In addition, *Clostridium* was positively associated with both somatic adipogenesis and visceral fat deposition in children (Awadel-Kariem et al., 2010), which is consistent with the genus *Clostridium* and its subordinate species, *C. spiroforme* and *C. celatum*, which were enriched with an increase in AFW and AFP, and a significantly higher abundance of *C. celatum* in the GG group after FMT. Moreover, the abundance of the genus *Parabacteroides* decreased as AFW and AFP increased, and then decreased in abundance after FMT, consistent with our previous finding that *Parabacteroides* and its species *Parabacteroides distasonis* were more enriched in lean than fat chickens (Xiang et al., 2021b). Previous studies reported that *P. distasonis* alleviates obesity and metabolic dysfunction by producing succinate and secondary bile acids (Wang et al., 2019).

Our metabolomic analyses revealed that alterations in the gut microbiota regulate fat deposition by affecting host metabolic profiles. Early transplantation of high-fat chicken gut microbiota changed the metabolite concentrations of many metabolites in the cecum content of Qingyuan partridge chickens. Under conditions of oxygen radical catalysis and a series of chemical reactions, arachidonic acid produces substances similar in structure to prostaglandins (Morrow et al., 1990). Saturated fatty acids are the main components of lipids and a variety of saturated fatty acids, such as lauric acid, molluscic acid, and stearic acid, are commonly found in agricultural animal fats. Unsaturated fatty acids are essential fatty acids for the host, and arachidonic acid converted from oleic acid is present mainly in the cell membrane (Xu, 1995). More arachidonic acid was observed in the recipients of high-fat chicken gut microbiota transplantation, which may be due to the fact that arachidonic acid alters the permeability of cell membranes and promotes chicken fat deposition (Zhou et al., 2021). Obese individuals can obtain more energy from food than lean individuals, potentially because of differences in their gut microbiome composition, and obese individuals may further stimulate adipogenesis after energy acquisition (Turnbaugh et al., 2006). These results, combined with our findings, suggest that the compounds produced by the gut microbiota during the regulation of unsaturated fatty acid concentration and amino acid metabolism affect lipid metabolism to increase abdominal fat deposition in chickens. Early FMT from the high-fat chickens also resulted in the differential accumulation of metabolites such as 2-amino-1,3,4-octadecanetriol, which has been shown to stimulate the synthesis of leptin in adipocytes and reduce excess subcutaneous fat when combined with lipolytic mixtures. Shikonin improves the reaction of oxygen free radicals with polyunsaturated fatty acids to form lipid peroxide by promoting neutrophil apoptosis (Zhang et al., 2020), while in 3 T3-L1 adipocytes, shikonin can inhibit the accumulation of triglycerides and the formation and deposition of fats, suggesting that shikonin has an anti-obesity effect (Lee et al., 2010). DL-carnitine is an amino acid-like compound that can promote the conversion of fat into energy in the body. It also has various physiological functions, such as oxidizing and decomposing fat, weight loss, and anti-fatigue, and is widely used as a food additive and nutritional supplement for humans and animals (Tao et al., 1981; Visser et al., 1986).

The gut microbiota and its metabolites can influence host gene expression, immune system development, and maintenance of intestinal physiological functions and, thus, participate in host lipid metabolism (Jing et al., 2022; Chen et al., 2023; Feng et al., 2023). Our previous study showed that multiple microbial taxa can regulate lipid biosynthesis and energy metabolism in the Qingyuan partridge chicken by producing secondary metabolites that enhance energy capture, promote fat deposition, or alleviate obesity (Xiang et al., 2021b). In the present study, the functional pathways of the differential microbial taxa between the GG and EE groups were primarily metabolic pathways. Early transplantation of high-fat gut microbiota upregulated the pathways of amino acid biosynthesis, primary bile acid biosynthesis, methane metabolism, and metabolic pathways, while the pathways of alpha linolenic acid metabolism, linoleic acid metabolism, and biosynthesis of terpenoids and steroids were downregulated. These findings are consistent with the results of the transplantation of fresh bacterial suspensions from adult high-fat poodles into the intestines of puppies (Tuniyazi et al., 2022). Both FMT research on dogs and Qingyuan partridge chickens demonstrated that early transplantation of high-fat gut microbiota enhanced a variety of metabolic processes, such as carbohydrate metabolism, lipid metabolism, and energy metabolism, as well as other metabolic processes of gut microbial metabolites (Cai et al., 2020). The present study also found that transplantation of high-fat gut microbiota had a significant effect on metabolite functions in the cecum of Qingyuan partridge chickens. Early transplantation of high-fat chicken gut microbiota significantly promoted lipid and peptide functions and reduced the biological functions related to vitamins and cofactors, nucleic acids, steroids, carbohydrates, hormones, and transmitters. These were consistent with previous studies that tryptophan a proper adding of tryptophan to the basic diet of broilers and laying hens can effectively affect abdominal fat deposition in chickens (Rosa et al., 2001; Fatufe et al., 2005) and therefore tryptophan is a common additive used in livestock farming to improve intestinal epithelial barrier through its metabolites (Liikonen et al., 2023; Fu et al., 2023b). L-phenylalanine, threonine, and tyrosine are metabolized to phenolic compounds and terpenoids by microbial activities, whereas amino acids such as proline, leucine, and isoleucine can be converted to fatty acids by further biochemical reactions (Nicholson et al., 2012).

## 5 Conclusion

Early transplantation of high-fat chicken gut microbiota did not significantly alter the growth, development, or carcass traits of recipient chickens, but did reduce the coefficient of variation of chicken abdominal fat deposition traits. Transplantation resulted in a significant decrease in the abundance and changes in the structure of the cecum microbiome and significantly altered the metabolite composition of chicken cecum contents by regulating the biological functions associated with energy metabolism and fat synthesis.

## Data availability statement

The datasets presented in this study can be found in online repositories. The names of the repository/repositories and accession number(s) can be found at: https://www.cncb.ac.cn/, CRA013128; https://www.cncb.ac.cn/, OMIX005134.

## Ethics statement

The animal studies were approved by Laboratory Animal Welfare and Animal Experimental Ethical Inspection board of Foshan University. The studies were conducted in accordance with the local legislation and institutional requirements. Written informed consent was obtained from the owners for the participation of their animals in this study.

## Author contributions

JS: Writing – original draft, Data curation, Formal analysis, Investigation, Validation, Visualization. CL: Writing – original draft, Data curation, Formal analysis, Investigation, Methodology, Visualization. ZL: Writing – original draft, Data curation, Formal analysis, Investigation. JL: Investigation, Resources, Supervision, Writing – review & editing. LX: Writing – original draft, Data curation, Investigation, Methodology. XZ: Writing – original draft, Formal analysis, Investigation, Visualization. ZX: Writing – original draft, Data curation, Investigation, Visualization. XL: Writing – original draft, Data curation, Investigation. ZM: Data curation, Formal analysis, Methodology, Writing – review & editing. JD: Investigation, Methodology, Resources, Writing – review & editing. HL: Writing – review & editing, Funding acquisition, Project administration, Resources, Supervision. HX: Writing – review & editing, Conceptualization, Formal analysis, Funding acquisition, Project administration, Resources, Supervision.

## Supplementary material

SUPPLEMENTARY FIGURE S1 The accumulating curve of body weight of GG and EE chickens.

SUPPLEMENTARY FIGURE S2 The Pearson’s correlation of the abundance of different enriched gut microorganism and the AFD of Qingyuan partridge chickens.

SUPPLEMENTARY FIGURE S3 Quality control of metabolites detection under positive **(A)** and negative **(B)** ion mode.

SUPPLEMENTARY FIGURE S4 The clustering heatmap of positive **(A)** and negative ion mode **(B)** metabolites.

SUPPLEMENTARY FIGURE S5 The SVM classifier of positive ion metabolites.

SUPPLEMENTARY FIGURE S6 The SVM classifier of negative ion metabolites.
