# Supplementary figures and images for: Early fecal microbiota transplantation from high abdominal fat chickens affects recipient cecal microbiome and metabolism
Source: Front Microbiol. 2024 Jan 8;14:1332230. doi: 10.3389/fmicb.2023.1332230 (PMC10800977; doi:10.3389/fmicb.2023.1332230)

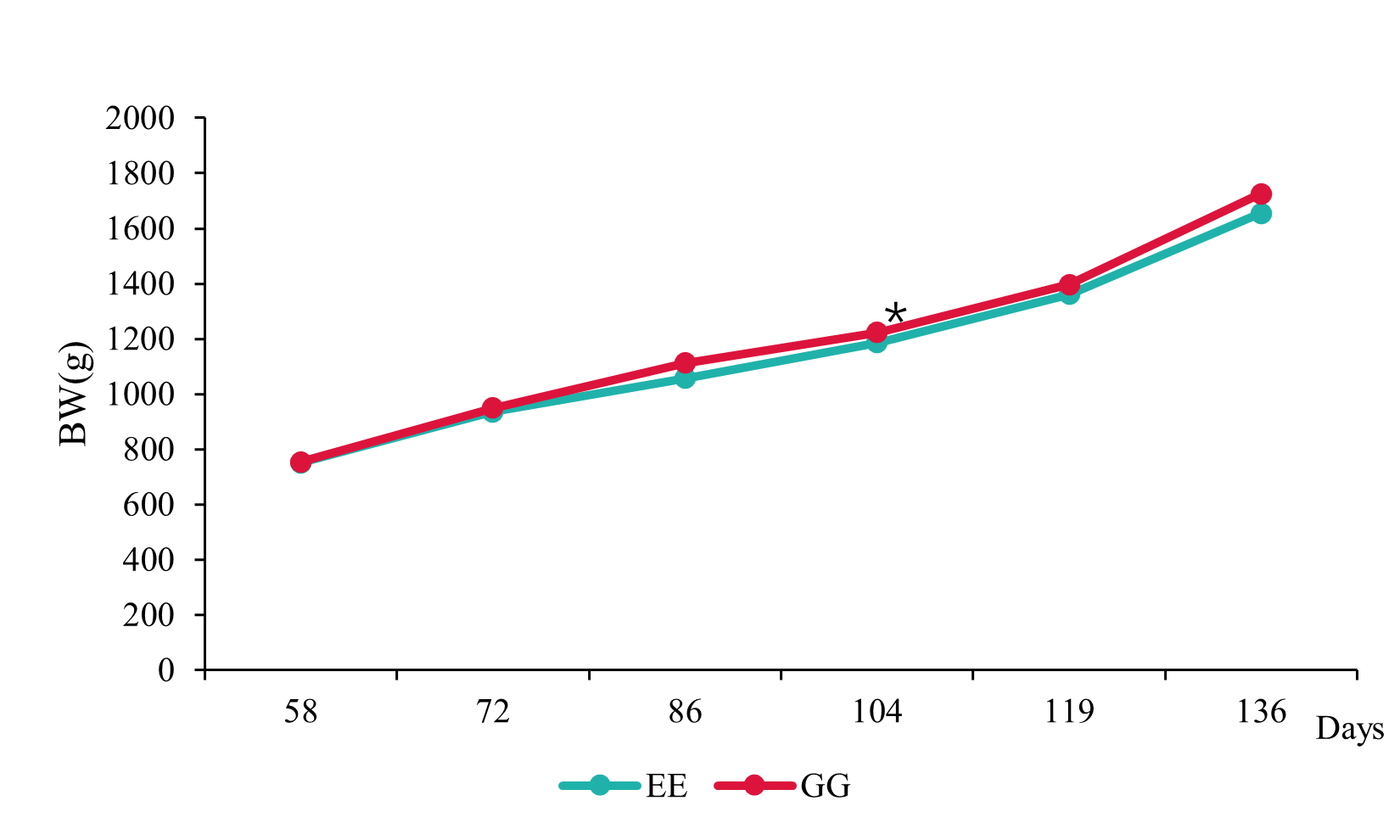

Supplement: SUPPLEMENTARY FIGURE S1 — The accumulating curve of body weight of GG and EE chickens. [file Image_1.jpeg]

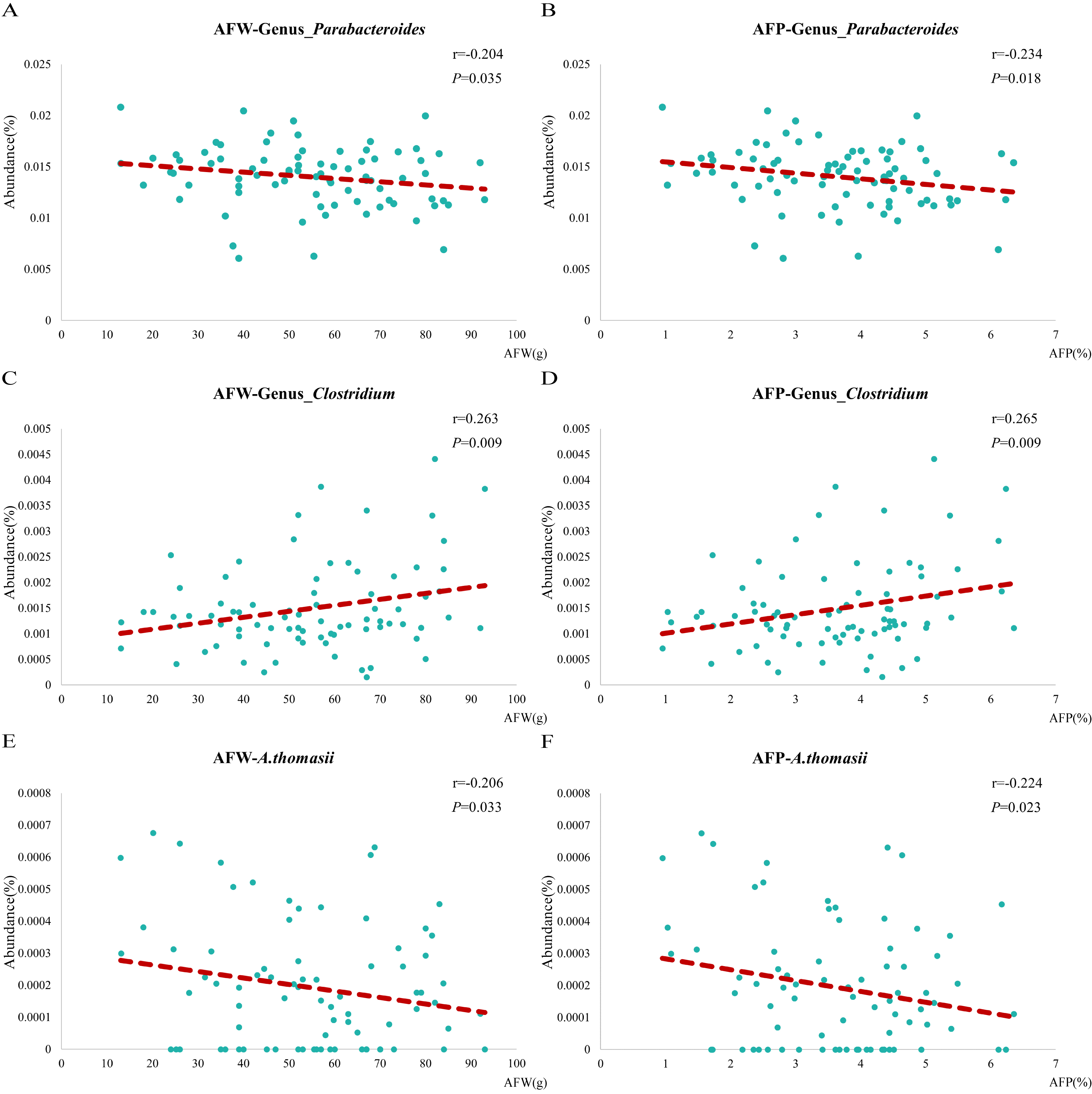

Supplement: SUPPLEMENTARY FIGURE S2 — The Pearson’s correlation of the abundance of different enriched gut microorganism and the AFD of Qingyuan partridge chickens. [file Image_2.jpeg]

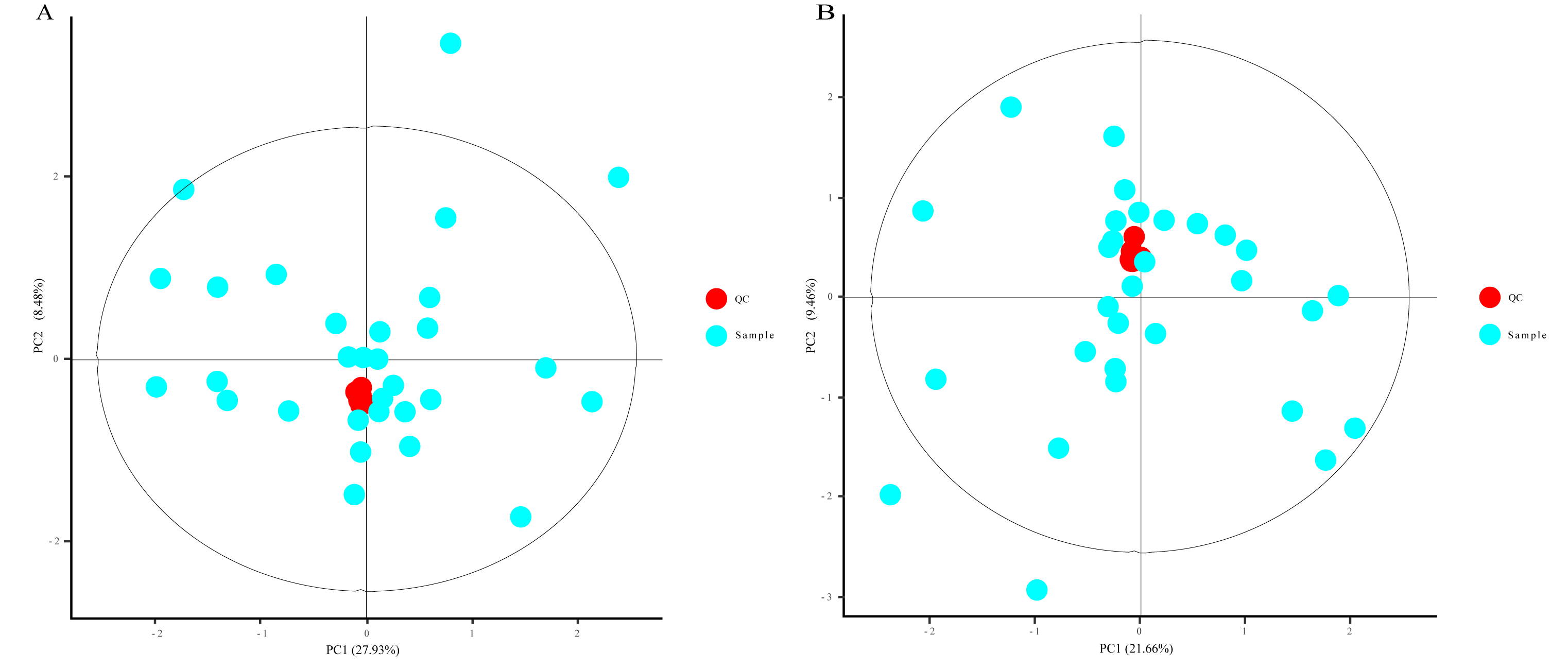

Supplement: SUPPLEMENTARY FIGURE S3 — Quality control of metabolites detection under positive (A) and negative (B) ion mode. [file Image_3.jpeg]

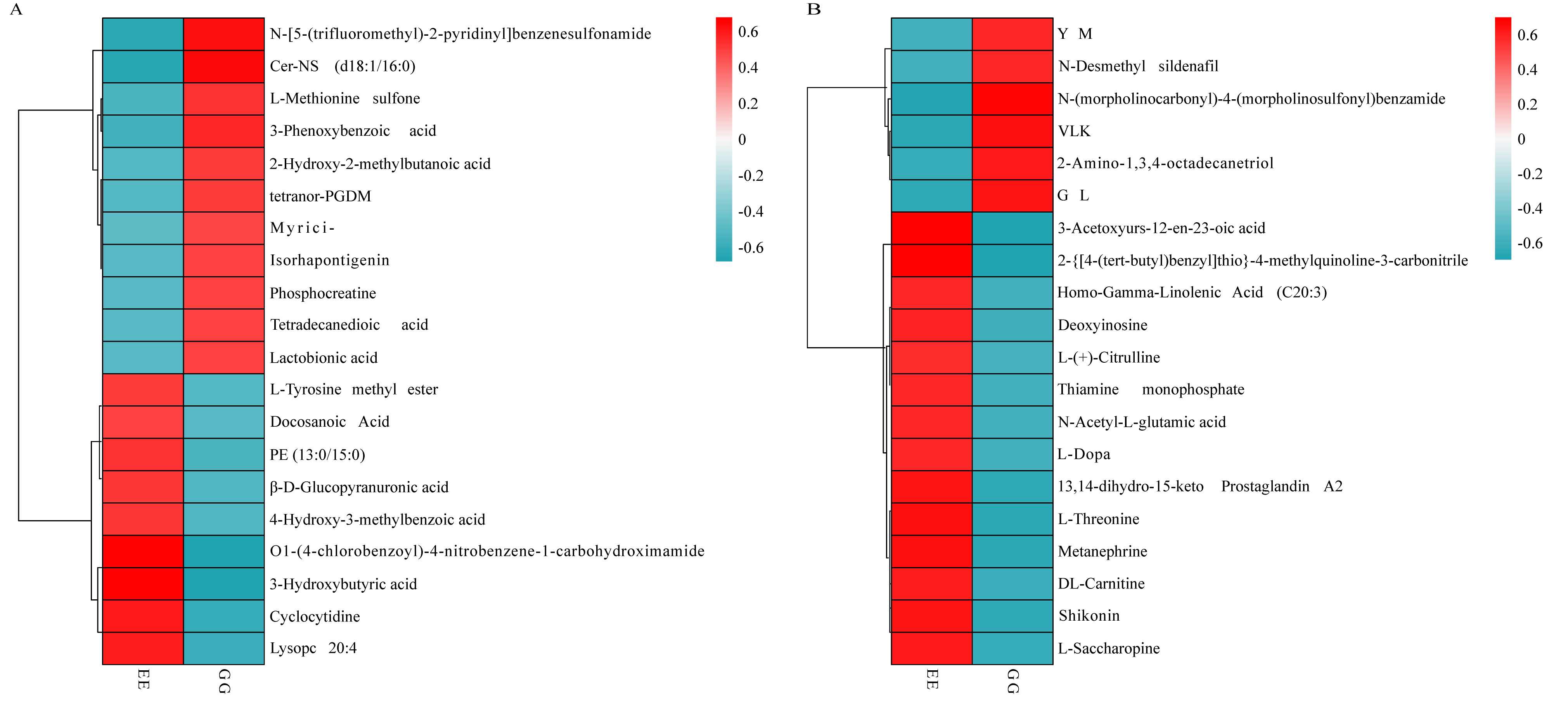

Supplement: SUPPLEMENTARY FIGURE S4 — The clustering heatmap of positive (A) and negative ion mode (B) metabolites. [file Image_4.jpeg]

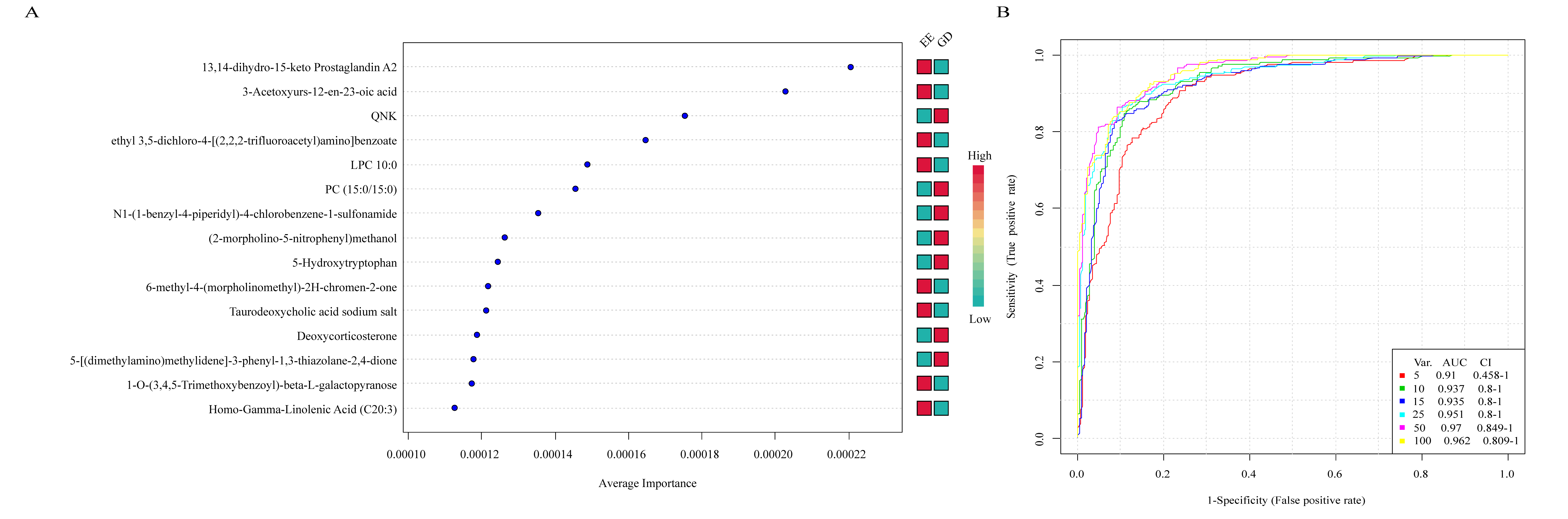

Supplement: SUPPLEMENTARY FIGURE S5 — The SVM classifier of positive ion metabolites. [file Image_5.jpeg]

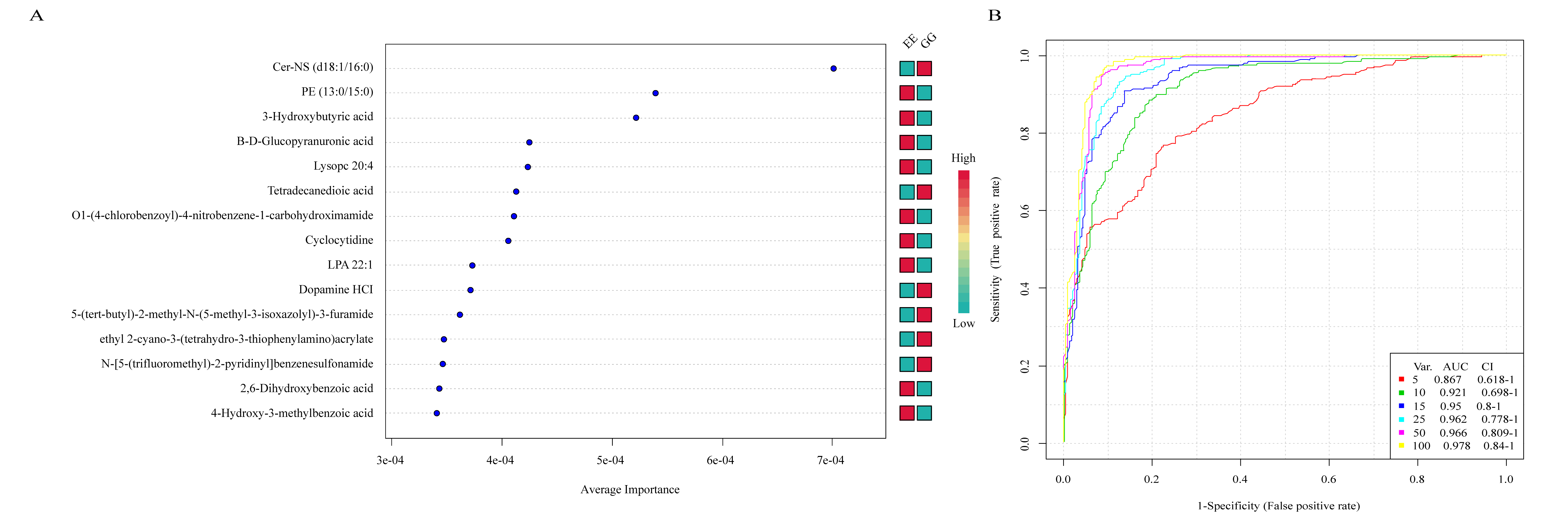

Supplement: SUPPLEMENTARY FIGURE S6 — The SVM classifier of negative ion metabolites. [file Image_6.jpeg]
